# Elevated Blood Pressure and Cardiac Mechanics in Children and Adolescents: A Systematic Review and Meta-Analysis

**DOI:** 10.1093/ajh/hpaf026

**Published:** 2025-02-20

**Authors:** Andrea Faggiano, Elisa Gherbesi, Carla Sala, Stefano Carugo, Guido Grassi, Cesare Cuspidi, Marijana Tadic

**Affiliations:** Department of Cardio-Thoracic-Vascular Diseases, Foundation IRCCS Ca’ Granda Ospedale Maggiore Policlinico, Milano, Italy; Department of Cardio-Thoracic-Vascular Diseases, Foundation IRCCS Ca’ Granda Ospedale Maggiore Policlinico, Milano, Italy; Department of Cardio-Thoracic-Vascular Diseases, Foundation IRCCS Ca’ Granda Ospedale Maggiore Policlinico, Milano, Italy; Department of Clinical Sciences and Community Health, University of Milano, Milano, Italy; Department of Cardio-Thoracic-Vascular Diseases, Foundation IRCCS Ca’ Granda Ospedale Maggiore Policlinico, Milano, Italy; Department of Clinical Sciences and Community Health, University of Milano, Milano, Italy; Department of Medicine and Surgery, University of Milano-Bicocca, Milano, Italy; Department of Medicine and Surgery, University of Milano-Bicocca, Milano, Italy; University Heart Center Ulm, University Ulm, Ulm, Germany

**Keywords:** blood pressure, global longitudinal strain, hypertension, left ventricular ejection fraction, pediatric hypertension, systolic function

## Abstract

**INTRODUCTION:**

Evidence on left ventricular (LV) mechanics, assessed by speckle tracking echocardiography (STE), in children and adolescents with elevated blood pressure (BP)/hypertension is scanty.

**AIM:**

The aim of the present meta-analysis was to provide an updated information on LV systolic function phenotyped by global longitudinal strain (GLS) and LV ejection fraction (LVEF) in the setting of pediatric hypertension.

**METHODS:**

Systematic searches were conducted across bibliographic databases (Pub-Med, OVID, EMBASE, and Cochrane Library) to identify eligible studies from inception up to 30 November 2024. Studies reporting data on LV mechanics in pediatric hypertension and controls were included. The statistical difference of the echocardiographic variables of interest between groups such as LVEF and GLS was calculated by standardized mean difference (SMD) with 95% confidence interval (CI) using random-effects models.

**RESULTS:**

Eight studies including 719 individuals with elevated BP/hypertension and 1,653 age-matched healthy controls were considered for the analysis. Pooled average LVEF values were 72.4 ± 1.6% in the healthy control group and 72.5 ± 1.8% in the elevated BP/hypertensive group (SMD: 0.08 ± 0.15, CI: −0.21/0.36, *P* = 0.60); the corresponding values of GLS were −19.6 ± 1.1% and 18.5 ± 0.9% (SMD: −0.96 ± 0.25, CI: −1.46/−0.47, *P* < 0.0001). A parallel impairment of global circumferential strain emerged from pooled data of three studies (SMD: −0.96 ± 0.25, CI: −1.46/−0.47, *P* < 0.0001).

**CONCLUSIONS:**

Our data suggest that LVEF is unable to detect early alterations in systolic function in pediatric hypertension, and the implementation of STE may be highly useful in unmasking systolic dysfunction in this setting.

Subclinical cardiac organ damage in children and adolescents with hypertension may develop early, and the reference phenotype for diagnosis is increased left ventricular mass (LVM) assessed by conventional transthoracic echocardiography.^1,2^ In the last decades, a large body of evidence has accumulated on this issue providing detailed information on the prevalence of LV hypertrophy (LVH) in the pediatric population. A recent international multicenter study including 990 hypertensive children aged ≤ 18 years, enrolled from four academic centers, found LVH in 29.1% of subjects with essential hypertension and in 36.5% of their counterparts with secondary hypertension.^3^ The association between different blood pressure (BP) phenotypes and the prevalence of LVH has been well documented in a cohort of 294 adolescents with ambulatory pre-hypertension and hypertension. The prevalence of LVH ranged from 10.3% in subjects with pre-hypertension to 20.0% with white coat hypertension and 32.0% with severe ambulatory hypertension.^4^ The above-mentioned studies, in line with the findings of many other authors, suggest that LV structural changes may appear early in a large fraction of children and adolescents with elevated BP. This was recently confirmed by a meta-analysis of fifteen studies which reported a prevalence range of 7.7-54%.^5^

Unlike the evidence that emerged on LV structural modifications, the vast majority of studies targeting LV systolic function in pediatric hypertension, assessed with LV ejection fraction (LVEF) or LV fractional shortening (FS) failed to show impairment of systolic performance.^6,7^ The meta-analysis by Rus *et al*.,^5^ based on thirteen studies evaluating LVEF, did not reveal any significant difference between hypertensive and normotensive individuals; however it showed higher values of FS in hypertensive patients (eleven studies), probably related to the hyperkinetic state of juvenile hypertension.

Deformation imaging (i.e., 2D-3D speckle tracking echocardiography) is increasingly used as a more advanced metric in measuring LV function compared to LVEF and FS in the diagnosis and management of several cardiac diseases in children.^8^ This angle and load-independent method for myocardial strain measurement may help to better define the real impact of hypertension on LV systolic function than conventional echocardiography in hypertensive children and adolescents.^9^ Starting from a consolidated consensus regarding the greater diagnostic sensitivity and prognostic value of LV mechanics, namely global longitudinal strain (GLS), in assessing LV systolic function, we performed a meta-analysis to expand the focus on this topic by providing an up-to-date and comprehensive information on changes of myocardial deformation in children and adolescents with high BP/hypertension.^10–12^

## METHODS

The review was performed according to the key recommendations provided by the Preferred Reporting Items of Systematic Reviews and Meta-Analyses (PRISMA) statement 2020,^13^ and prospectively registered with the International Prospective Register of Systematic Reviews (ID: CRD42024621213). Medical literature was reviewed in order to identify all articles evaluating LV mechanics by STE in children/adolescents with elevated BP/hypertension. To this purpose, a systematic search was performed using four electronic databases (Pub-Med, OVID, EMBASE, and Cochrane Library) from inception up to 30 November 2024. Searches were limited to clinical investigations published in English. Studies were identified by using MeSH terms and crossing the following search items: “pediatric hypertension,” “elevated blood pressure,” “heart,” “cardiac disease,” “myocardial strain,” “left ventricular mechanics,” “longitudinal global strain,” “speckle tracking echocardiography,” “systolic dysfunction,” “left ventricular ejection fraction.”

Checks of the reference lists of original papers and pertinent review articles were also searched for additional relevant literature. Data were examined and extracted by three independent investigators (E.G., A.F., and C.C.). In case of no agreement on a specific record, the full text of the study was analyzed by all reviewers in order to establish its eligibility according to the inclusion criteria mentioned below.

The main inclusion criteria were: (i) English articles published in peer-reviewed journals; (ii) studies providing data on GLS by STE in children or adolescents (excluding infants or children under three years of age) with elevated BP/hypertension and preserved LVEF compared to healthy normotensive individuals; (iii) minimum set of clinical/demographic data (i.e., sex and age).

Two independent investigators based on the Newcastle-Ottawa Scale (http://www.ohrica/programs/clinical_epidemiologyoxford.html) assessed the methodological quality of each study (C.C. and E.G.). The Newcastle-Ottawa Scale of seven or more was considered as a good quality.

### Statistical analysis

The primary aim of the meta-analysis was to compare LV systolic function assessed by GLS in children and adolescents with hypertension/elevated BP and preserved LVEF with that of their healthy normotensive counterparts. Additional conventional echocardiographic parameters were also considered in the analysis (see Results below).

A pooled analysis of demographic and clinical variables was performed using fixed or random effects meta-analysis by Comprehensive Meta-Analysis Version 2, BioStat, Englewood, NJ. Standardized mean difference (SMD) with 95% confidence interval (CI) was calculated to test the statistical difference of continuous echocardiographic variables between healthy controls and individuals with elevated BP/hypertension.

Demographic, clinical, and echocardiographic data provided by selected studies were expressed as absolute numbers, percentages, mean ± SD, mean ± SE, or mean with CI.

The random effect model was applied due to the high heterogeneity across studies (I^2^ >75). To assess the effect of individual studies on the pooled result, we conducted a sensitivity analysis by excluding each study one by one and recalculating the combined estimates on the remaining studies. Meta-regression analysis was used to test the linear relationship between GLS, LV mass index (LVMI), and systolic BP.

Publication bias was assessed by using the funnel plot method (Trim and fill test) Statistical significance was set at *P* < 0.05.

## RESULTS

### Search results

**Figure 1.**
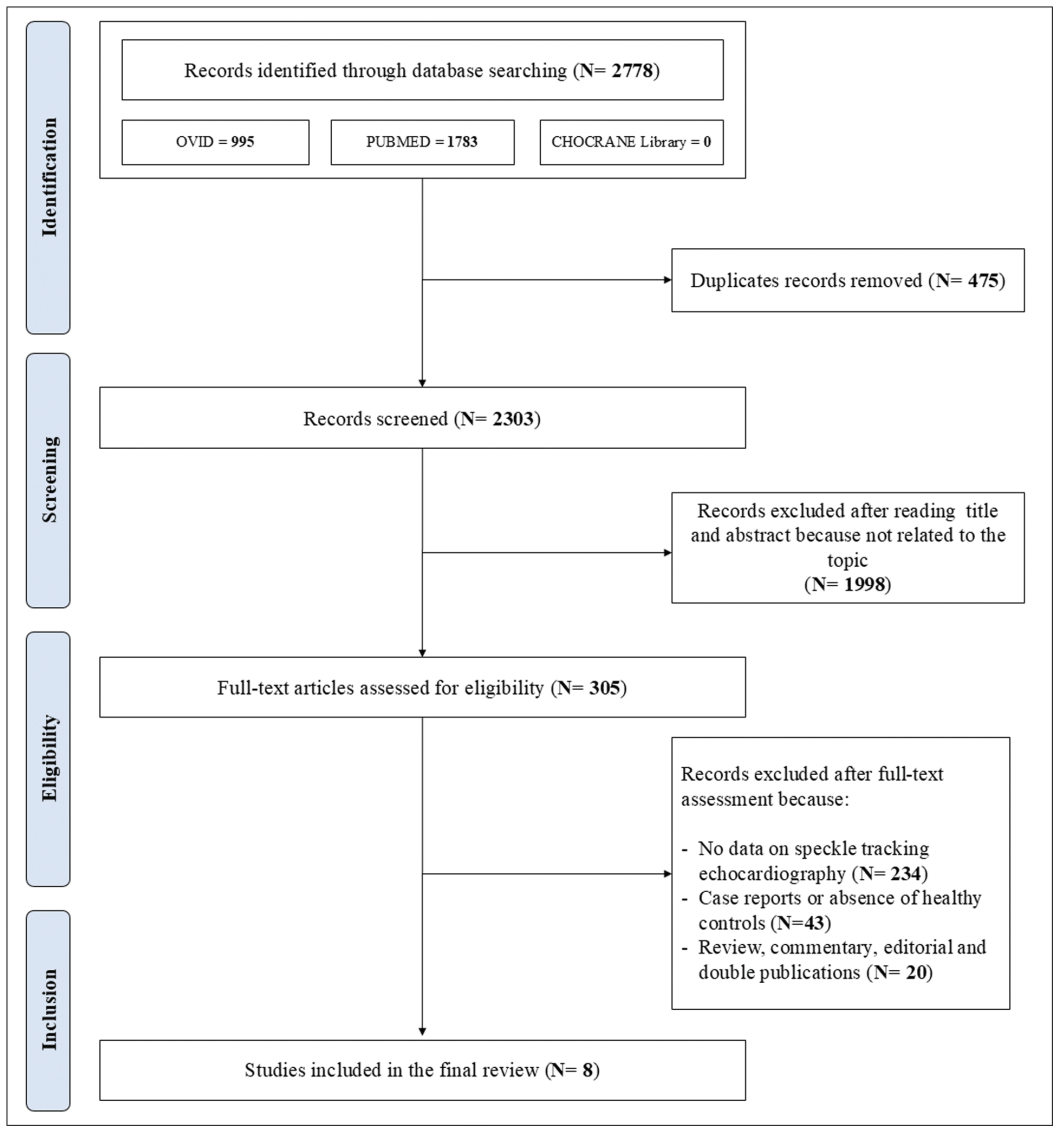
Schematic flowchart for the selection of studies.

The PRISMA flowchart as presented in **Figure 1** describes the full selection process. After removing duplicates, the first literature search identified 2,303 papers. After the initial screening of titles and abstracts, 1,998 studies were excluded as they were not related to the topic. Therefore, 305 studies were reviewed; of these, 234 did not report data on echocardiographic speckle tracking parameters, 43 were case reports or did not include healthy controls, 20 were review, commentary, editorial articles, and double publications. Thus, a total of eight studies focusing on LV mechanics (i.e., GLS) were included in the analysis.^14–20^ The Newcastle-Ottawa Score, used for assessing the quality of the studies, ranged from 7 to 9, with the mean score being 7.6. Therefore, no study was excluded based on its limited quality.

### Descriptive features of the studies

Seven hundred nineteen children/adolescents with elevated BP/hypertension and 1,653 age-matched healthy controls were included in eight studies (hypertensive sample size ranging from 19 to 191 participants) performed in three countries (China = 3; United States  = 3; United Kingdom = 2). Hypertension was essential in the vast majority of cases, only two studies included a fraction of approximately 38% and 35% of hypertensives with reno-vascular or reno-vascular hypertension.^18,21^

**Table 1** shows the demographic, clinical, and characteristics of participants from selected studies such as BP measurement methodology, setting, definition of elevated BP/hypertension, distribution by sex, sample size, mean age, body mass index (BMI), office systolic and diastolic BP. In six of the eight studies that provided this type of indication, BP was detected in a sitting position (two to four measurements) with the auscultatory method in four and with a validated electronic device in two studies.

**Table 1.** Summary of eight studies targeting left ventricular systolic function, as assessed by LV mechanics, in pediatric hypertension

Author, publication year	Setting	BP measurement methodology	Sample size (n)		M/F		Age (years)		BMI (kg/m^2^) BSA (m^2^)		Office SBP (mmHg)		Office DBP (mmHg)	
		**Device**	**HTN**	**Controls**	**HTN**	**Controls**	**HTN**	**Controls**	**HTN**	**Controls**	**HTN**	**Controls**	**HTN**	**Controls**
Navarini, 2017^14^	Hypertension defined as SBP, and/or DBP ≥ 95th percentile	Three sitting BP measurements Aneroid instrument	26	37	14/12	15/22	14.3 ± 2.4	11.4 ± 3.3	25.8 ± 5.8	19.9 ± 4.5	130 ± 16	104 ± 14	66 ± 15	55 ± 13
Zhang, 2018^15^	Hypertension defined according to ANHBPEPWG	Two sitting BP measurements Auscultatory method	19	195	11/8	113/82	9.7 ± 1.2	9.6 ± 1.2	17.3 ± 3.2	16.9 ± 3.0	125 ± 7	106 ± 7	79 ± 8	70 ± 7
Luo, 2018^16^	Masked hypertension	Three sitting BP measurements Validated electronic device	40	40	29/40	29/40	18.0 ± 2.6	18.3 ± 2.9	24.0 ± 5.0	20.0 ± 3.0	118 ± 9	112 ± 9	69 ± 7	64 ± 7
Tran, 2020 ^17^	Elevated BP SBP ≥ 90th percentile	Four sitting BP measurements Aneroid instrument	119	144	70/49	73/71	15.1 ± 1.8	15.6 ± 1.5	29.3 ± 7.6	26.2 ± 6.8	133 ± 7	111 ± 10	86 ± 9	75 ± 10
Gu, 2021^18^	Elevated SBP, and/or DBP ≥ 95th percentile	Three sitting BP measurements Aneroid instrument	81	47	50/81	22/25	14.3 ± 2.7	13.6 ± 2.9	24.4 ± 5.1	20.5 ± 4.7	130 ± 16	107 ± 13	67 ± 15	61 ± 10
Kaplinski, 2021^19^	Hypertension defined according AAP	n.a.	155	57	n.a.	n.a.	n.a.	n.a.	n.a.	n.a.	n.a.	n.a.	n.a.	n.a.
Wu, 2022^20^	Elevated BP SBP ≥ 90th percentile	Two sitting BP measurements Validated electronic device	191	1081	98/93	538/543	4 years	4 years	15.9 ± 0.96	14.6 ± 1.06	107 ± 4	94 ± 6	62 ± 7	56 ± 5
Zhan, 2022^21^	Hypertension defined according AAP	n.a.	88	52	57/31	32/20	15.4 ± 2.9	14.5 ± 2.0	1.90 (1.56; 2.15)	1.66 ± 0.24	136 (131;143)	119 ± 12	68 (63;79)	68 ± 10

Abbreviations: AAP, American Academy of Pediatrics; ANHBPEPWG, American National High Blood Pressure Education Program Working Group on High Blood Pressure in Children and Adolescents; BMI, body mass index; BP, blood pressure; BSA, body surface area; DBP, diastolic blood pressure; HTN, hypertension; SBP, systolic blood pressure.

Data are shown as median (interquartile range) or mean ± SD.

Mean age ranged from four to eighteen years both in cases and in controls. The corresponding ranges values for BMI, office systolic, and diastolic BP were as follows: 15.9-26.0 kg/m^2^, 107-136 mmHg, 62-86 mmHg, in cases, and 14.6-26.2 kg/m^2^, 94-112 mmHg, 56-75 mmHg in controls, respectively. **Table 2** reports the pooled data of age, height, BMI, systolic and diastolic office BP. Systolic/diastolic BP values and BMI were significantly higher in cases than in controls, while age and height were not different between the two groups.

**Table 2.** Demographic and clinical characteristics of the pooled healthy normotensive controls and individuals with elevated BP/hypertension

Variables	N studies	Mean values ± SE healthy controls	Lower-upper limit Healthy controls	Mean values ± SE Individuals with elevated BP/HTN	Lower-upper limit Individuals with elevated BP/HTN	SMD, *P* value
Age (years)	7	12.4 ± 2.2	8.0-16.8	12.9 ± 2.8	7.5-18.4	−0.47 ± 0.33, *P* = 0.15
BMI (kg/m^2^)	6	19.6 ± 1.4	16.9-22.4	22.6 ± 2.8	17.1-28.2	0.61 ± 0.12, *P* < 0.001
Height (cm)	5	1.4 ± 0.13	1.2-1.7	1.5 ± 0.2	1.2-1.8	0.04 ± 0.06, *P* = 0-52
SBP (mmHg)	7	107.8 ± 3.8	100.2-115.2	125.7 ± 6.3	113.4-138.1	1.89 ± 0.24, *P* < 0.001
DBP (mmHg)	7	64.1 ± 3.7	57.0-71.3	71.3 ± 4.2	63.1-79.6	0.82 ± 0.16, *P* < 0-001

Abbreviations: BMI, body mass index; BP, blood pressure; DBP, diastolic blood pressure; HTN, hypertension; SBP, systolic blood pressure; SMD, standard mean difference.

### Echocardiographic findings and meta-analysis results

In all studies, the conventional analysis of cardiac structure and function was performed according to the recommendations of the American Society of Echocardiography and the European Association of Cardiovascular Imaging published in 2015. Left ventricular myocardial deformation was measured offline from 2D or 3D echocardiographic images using commercial dedicated softwares. R-R gating was used for LV strain assessment. LV endocardium was manually traced and corrected, if necessary, and average longitudinal strain curve was automatically provided by the software.

**Table 3** summarizes information on the echocardiographic device, the method used to evaluate the strain and, when available, the software used for its analysis, as well as data regarding conventional and strain echocardiographic parameters such as mean LVMI (seven studies), LVEF (six studies), E/e′ ratio (five studies), GLS (eight studies), GCS (three studies). Left ventricular mechanics was assessed by 2D STE in six studies and by 3D STE in two studies. Average values of LVEF varied from 56.5 to 67.6% in cases and from 58.5 to 66.6% in controls; the corresponding GLS values ranged from −15.1 to −23.6% and −18.5 to −23.7%, respectively.

**Table 3.** Methodological features and echocardiographic data of eight studies targeting left ventricular systolic function, as assessed by LV mechanics, in pediatric hypertension

Author, publication year	Echocardiography	LVMI g/h^2.7^		E/e′ ratio		LVEF (%)		GLS (%)	
		**HTN**	**Controls**	**HTN**	**Controls**	**HTN**	**Controls**	**HTN**	**Controls**
Navarini, 2017^14^	PhilipsIE33 ultrasound system. 3D STE; TomTec, software.	37.0 ± 12.2	28.92 ± 8.3	6.6 ± 1.5	5.5 ± 1.1	63.4 ± 3.3	63.4 ± 5.7	−15.1 ± 3.3	−18.5 ± 1.9
Zhang, 2018^15^	Artida; Toshiba Medical Systems. 3D STE	n. a.	n. a.	n. a.	n. a.	n.a.	n.a.	−15.8 ± 1.6	−19.5 ± 2.1
Luo, 2018^16^	Vivid E9 system; 2D STE EchoPAC V201 software	28.6 ± 6.5	25.4 ± 5.8	7.2 ± 1.7	6.7 ± 1.4	63.0 ± 4.0	61.0 ± 5.0	−18.0 ± 1.8	−18.9 ± 1.7
Tran, 2020^17^	2D STE TomTec software	34.0 ± 7	31.0 ± 7.0	6.5 ± 1.5	6.0 ± 1.4	56.5 ± 6.7	58.5 ± 7.1	−20.1 ± 3.3	−21.2 ± 3.3
Gu, 2021^18^	Philips IE33 or EPIQ ultrasound system 2D STE	25.5 ± 4.9	33.6 ± 3.2	6.2 ± 1.2	5.4 ± 0.9	60.1 ± 6.0	62.4 ± 5.0	−18.2 ± 2.6	−18.8 ± 2.6
Kaplinski, 2021^19^	GE ultrasound system 2D STE TomTec software	37.9 ± 10.3	34.3 ± 7.6	6.2 ± 1.2	7.2 ± 2.0	n.a.	n.a.	−18,6 ± 2.2	−18.2 ± 2.2
Wu, 2022^20^	EPIQ 7C; Philips Healthcare, 2D STE QLAB version 10.5 software	25.8 ± 4.6	26.6 ± 4.8	n. a.	n. a.	67.6 ± 4.6	66.6 ± 4.0	−23.6 ± 2.4	−23.7 ± 2.3
Zhan, 2022^21^	GE Vivid E95, 2D STE, EchoPAC software	43.5 (36.3; 51.0)	25.5 ± 6.2	n. a.	n. a.	64.4 ± 4.8	62.3 ± 3.4	−18.90 (−20.5; −17.2)	−18.2 ± 1.9

Abbreviations: E/e′, ratio of trans-mitral flow velocity to annular velocity; GLS, global longitudinal strain; HTN, hypertension; LV, left ventricular; LVEF, left ventricular ejection fraction; LVMI, left ventricular mass index .

Data are shown as median (interquartile range) or mean± SD.

### LV function

**Figure 2.**
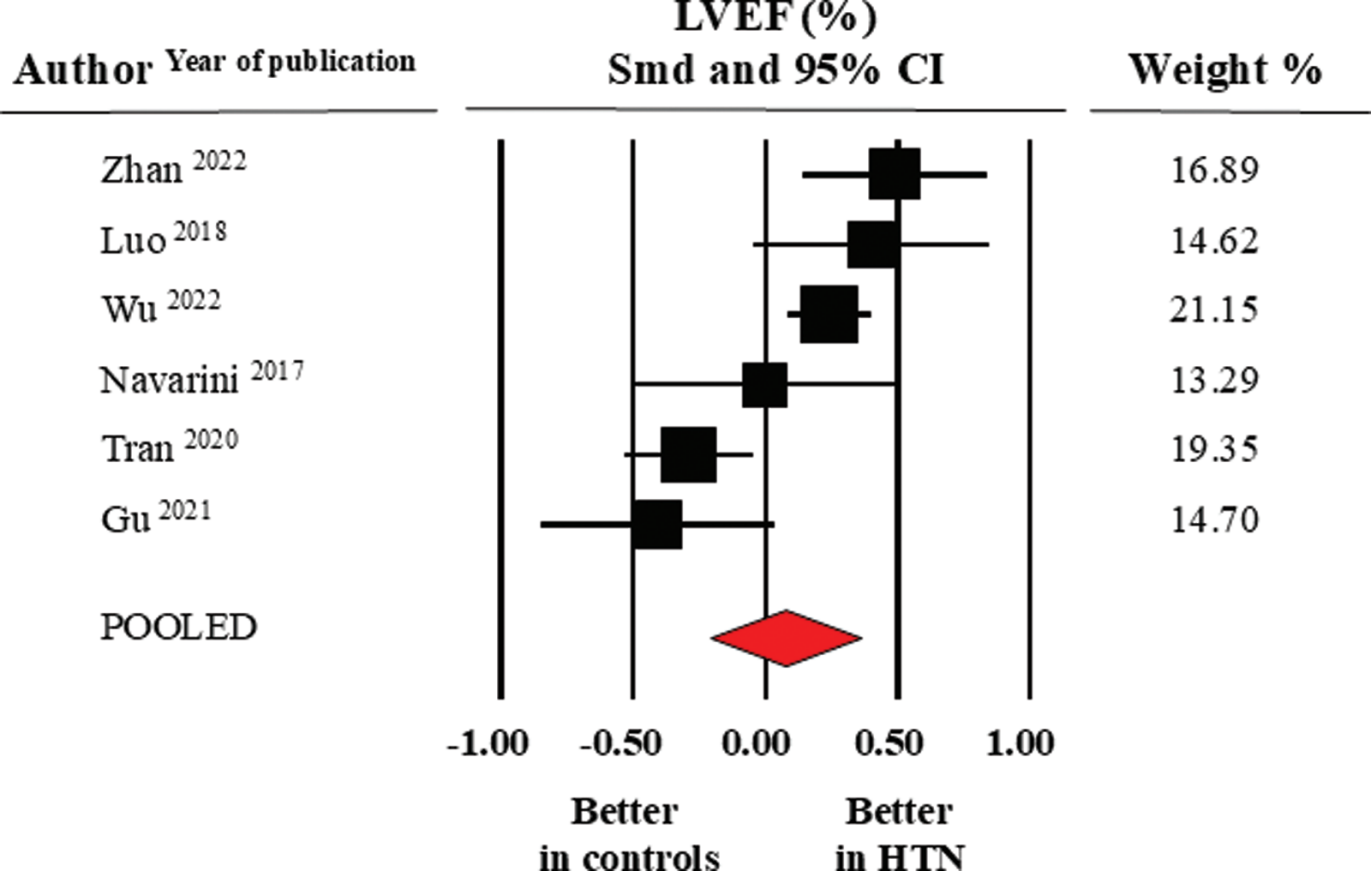
Forest plot of left ventricular ejection fraction (LVEF) in healthy normotensive controls and elevated BP/hypertensive individuals. Standard mean difference (SMD) and 95% confidence interval (CI); random model (I^2^ > 75%). BP, blood pressure.

Pooled average LVEF values were 72.4 ± 1.6% in the healthy control group and 72.5 ± 1.8 % in the elevated BP/hypertensive group. As depicted by the forest plot in **Figure 2**, the meta-analysis of selected studies did not reveal a significant difference between groups (SMD: 0.08 ± 0.15, CI: −0.21/0.36, *P* = 0.60).

**Figure 3.**
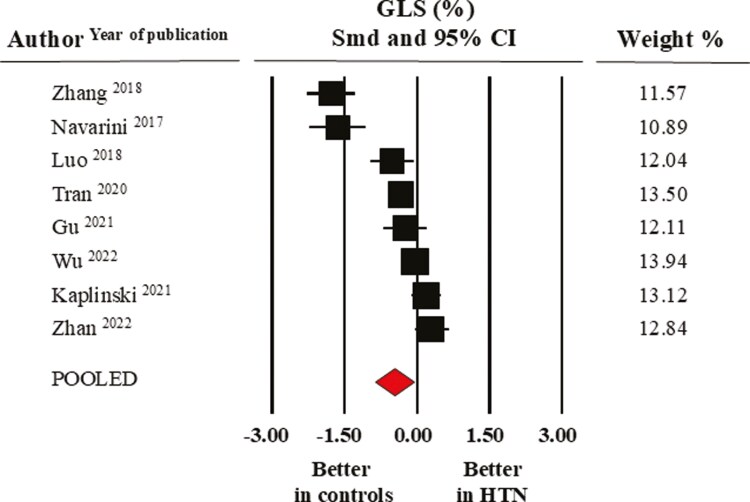
Forest plot of global longitudinal strain (GLS) in healthy normotensive controls and elevated BP/hypertensive individuals. Standard mean difference (SMD) and 95% confidence interval (CI); random model (I^2^ > 75%).

**Figure 4.**
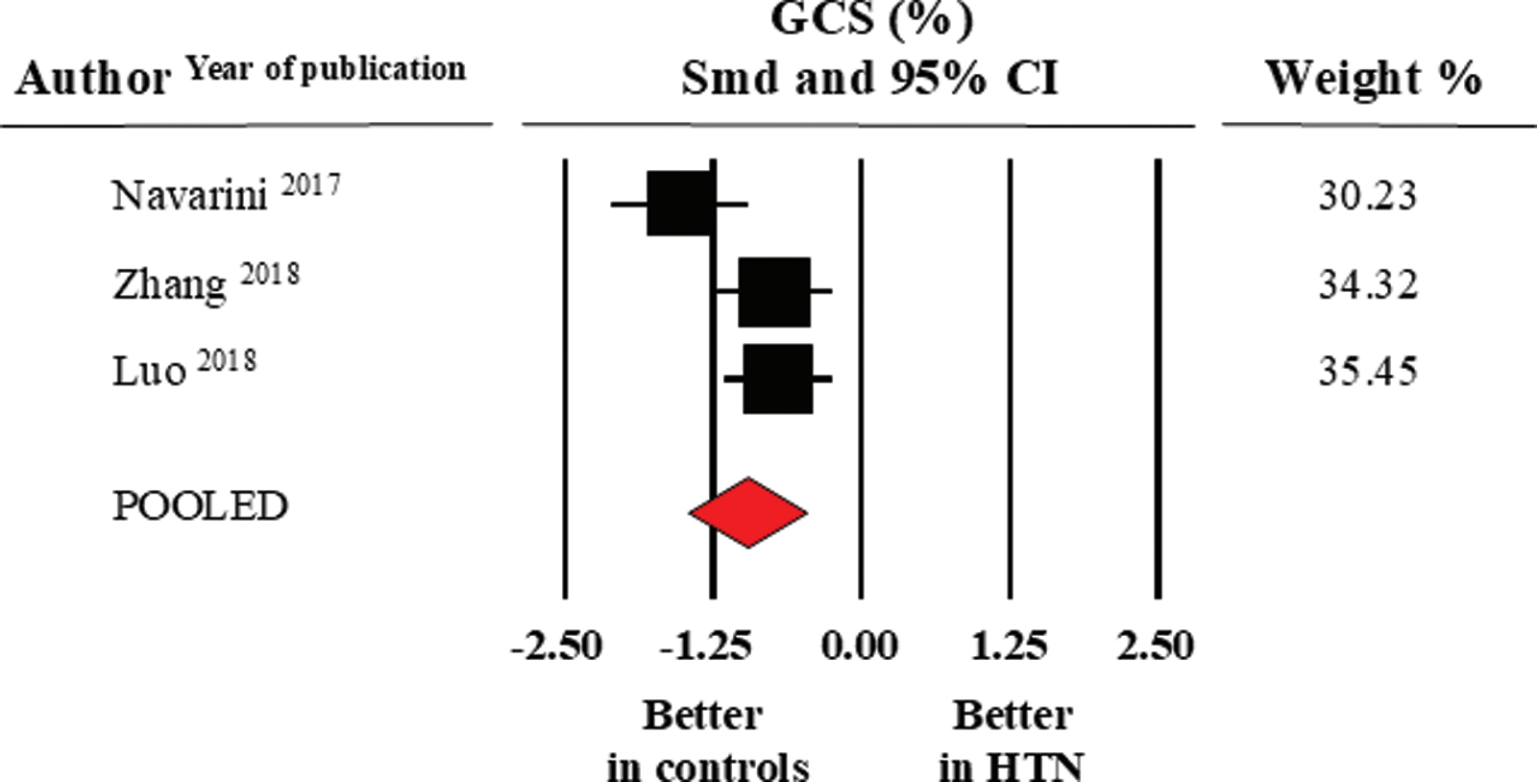
Forest plot of global circumferential strain (GCS) in healthy normotensive controls and elevated BP/hypertensive individuals. Standard mean difference (SMD) and 95% confidence interval (CI); random model (I^2^ > 75%). BP, blood pressure.

Pooled mean GLS values were −19.6 ± 1.1% in the control and −18.5 ± 0.9% in the elevated BP/hypertensive group. **Figure 3** shows the results of the meta-analysis of eight studies where SMD indicated that this index of systolic function was significantly lower in the elevated BP/hypertensive group (−0.46 ± 0.20, CI: −0.86/−0.07, *P* = 0.02). In a further meta-analysis restricted to three studies reporting data on GCS, the pooled mean values were −21.3 ± 1.0% in normotensive controls and −18.3 ± 1.5% in their hypertensive counterparts (SMD: −0.96 ± 0.25, CI: −1.46/−0.47, *P* < 0.0001) (**Figure 4**).

**Figure 5.**
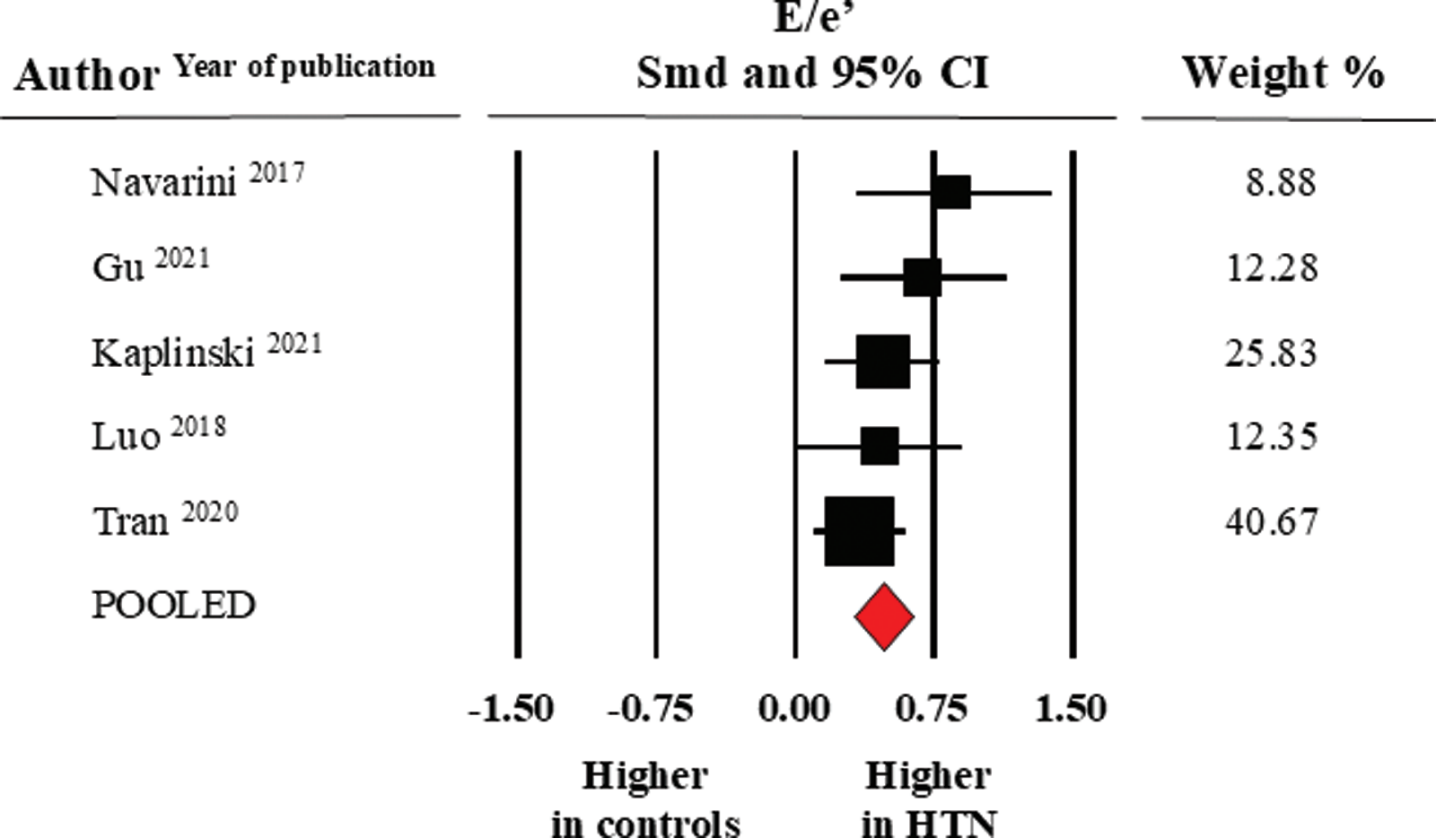
Forest plot of E/e′ ratio healthy normotensive controls and elevated BP/hypertensive individuals. Standard mean difference (SMD) and 95% confidence interval (CI); random model (I^2^ > 75%). BP, blood pressure.

As for LV diastolic function, as assessed by the E/A ratio (data from five studies), the average value was lower in elevated BP/hypertensive individuals (1.96 ± 0.11) than in normotensive controls (2.14 ± 0.13) with a significant SMD of −0.32 ± 0.06, CI: −0.43/−0.21, *P* = 0.0001. The value of the E/e′ ratio (data from five studies) was significantly greater in the pooled elevated BP/hypertensive group (6.93 ± 0.33) than in the control group (6.13 ± 0.28) with an SMD of 0.48 ± 0.08, CI: 0.32/0.64, *P* < 0.0001) (**Figure 5**).

### LV structure

**Figure 6.**
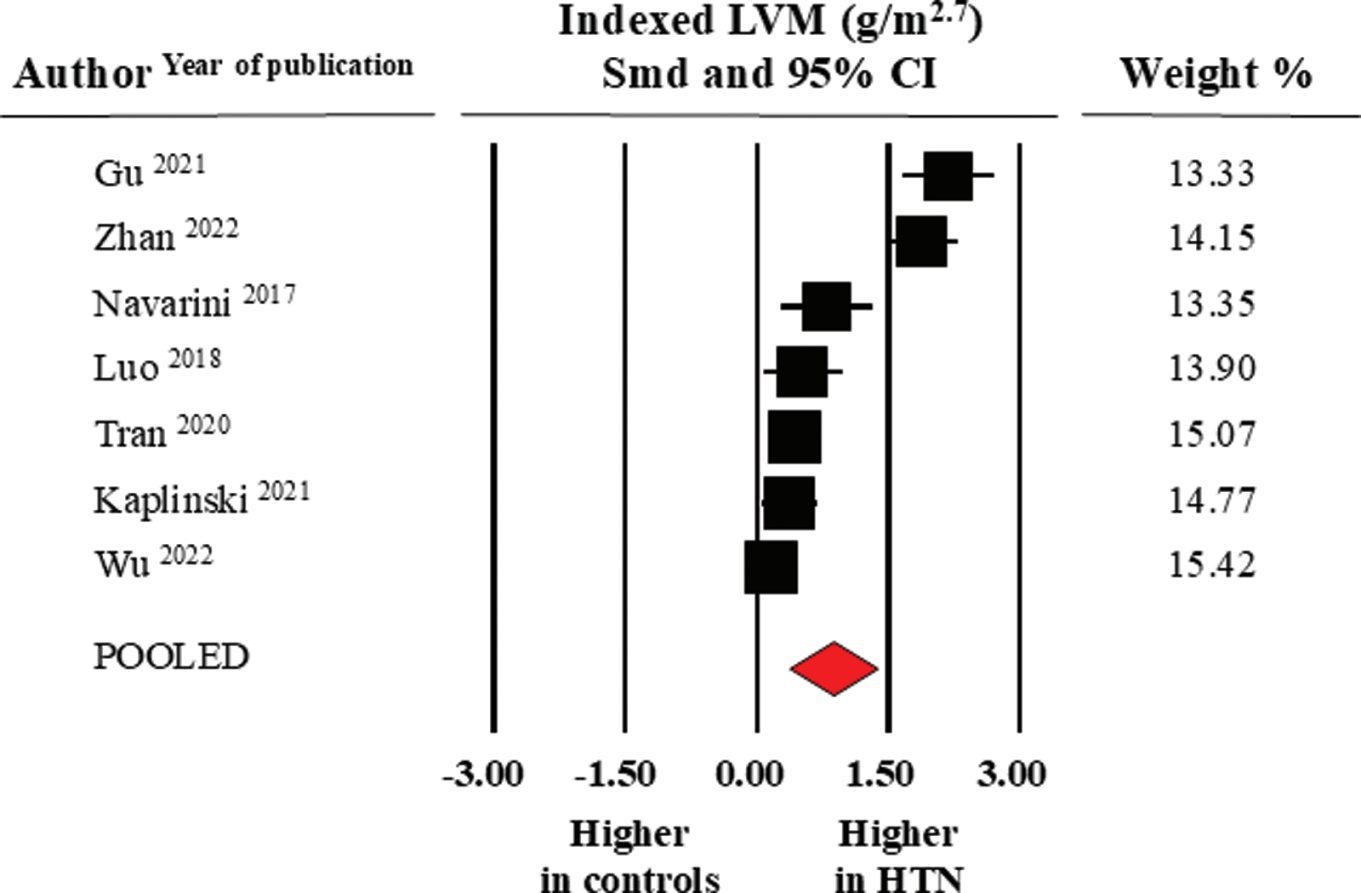
Forest plot of left ventricular mass index (LVM g/m^2.7^) in healthy normotensive controls and elevated BP/hypertensive individuals. Standard mean difference (SMD) and 95% confidence interval (CI); Random model (I^2^ >75%).

Pooled mean absolute LVM index (LVMI) values (data from seven studies) were 28.0 ± 1.2 g/h^2.7^ in normotensive controls and 34.4 ± 2.0 g/h^2.7^ in elevated BP/hypertensive individuals (SMD: 0.88 ± 0.25, CI: 0.38/1.38, *P* < 0.0001) (**Figure 6**).

### Publication bias

No publication bias was observed for all the analyses performed in the study. **Supplementary Figure S1** shows the funnel plot assessing publication bias for SMD of GLS in individuals with elevated BP/hypertension and controls. No single study effect was observed for the analysis of LVEF, GLS, and LVMI.

### Additional analyses

The analysis restricted to six studies that included participants aged fourteen to eighteen years revealed that GLS was lower in the elevated BP/hypertensive group (SMD: −0.20 ± 0.07, CI: −0.34/−0.06, *P* = 0.006) (**Supplementary Figure S2**). This was not the case for LVEF (SMD: −0.02 ± 0.08, CI: −0.18/0.14, *P* = 0.78).

### Meta-regression

The meta-regression of GLS on systolic BP showed a significant inverse correlation between the two parameters (coefficient = −0.137 ± 0.048, CI: −0.231/−0.043, *P* = 0.004 (**Supplementary Figure S3**). On the contrary, the meta-regression of LVMI on systolic BP highlighted a direct relationship between the two variables (coefficient = 0.246 ± 0.048, CI: 0.152/0.340, *P* < 0.0001) (**Supplementary Figure S4**).

## DISCUSSION

The value of echocardiographic assessment of LV remodeling in hypertensive children and adolescents is often underestimated. This is because the hyperdynamic status in hypertensive children and adolescents can actually mask early functional alterations leading to the development of overt organ damage which in turn results in CV disease later in life. Therefore, identifying subclinical cardiac damage is of great importance in the pediatric setting in order to make appropriate clinical and therapeutic decisions aimed at preventing adverse CV outcomes in the following decades.

Previous studies carried out in hypertensive children and adolescents showed increased LVMI and diastolic dysfunction, highlighting a correlation between BP values and the extent of deterioration in LV structure and diastolic function, without identifying, however, a coexisting systolic dysfunction.^5–9^ In line with these previous observations, our meta-analysis, through meta-regression, showed a direct relationship between LVMI and systolic BP. To date, the value of LV mechanics in detecting subclinical damage in the pediatric setting has not been extensively investigated and most studies had a limited number of participants.^14,15,17^ Thus, the present meta-analysis extends the knowledge on this topic showing that not only that LVMI was higher in hypertensive patients and LV diastolic function was impaired, but, more importantly, LV global longitudinal and circumferential strains were significantly lower in the pooled population. Strain reduction, even though in the normal range, might be a good indicator of adverse outcomes in young patients. There are several hypotheses to explain the deterioration of LV mechanics in young hypertensive patients. LVH is the most widely accepted structural substrate of LV strain reduction. However, even in patients with normal LVMI and hypertension, as well as with pre-hypertension, strain might be reduced.^22^ This can be explained by interstitial myocardial fibrosis and inflammation triggered by overactivation of the sympathetic nervous system, oxidative stress, impaired sodium handling, and hemodynamic changes in young hypertensive patients.^23^ The last two mentioned mechanisms can induce mechanical changes independently of interstitial changes. It has been documented that children with arterial hypertension have hyperkinetic LV characterized by increased FS, elevated LVEF, cardiac output, and normal total peripheral resistance.^24,25^ However, long-term hypertension and higher BP levels can result in elevated peripheral resistance and converting hemodynamics from a “cardiac” phenotype presented as a hyperkinetic LV to a “vascular” phenotype with less pronounced elevation or even normalization of cardiac output and increased peripheral resistance.

The high prevalence of obesity and metabolic syndrome in hypertensive young patients cannot be neglected because they do not exert only additive, but synergistic negative effects on LV structure, function, and mechanics.^5^ Navarini *et al*. showed that BMI correlated with LV 2D GCS, but not with longitudinal and radial strains in hypertensive children and adolescents.^14^ Zhang *et al*. reported that BMI, total cholesterol, and triglyceride levels correlated well with 3D longitudinal and circumferential strains.^15^ Therefore, we cannot exclude that the deterioration in LV mechanics documented by our meta-analysis in the elevated BP/hypertensive pooled group was partly influenced by the difference in BMI compared to healthy controls (+3 kg/m^2^). Similarly, differences in loading conditions (namely after-load) may have contributed to GLS reduction in the hypertensive setting independently of LV structural and functional changes. Indeed, our meta-regression highlighted a significant inverse relationship between GLS and systolic BP.

Despite this limitation, the present meta-analysis provides an important new piece of information showing that both, GLS and GCS were significantly reduced in hypertensive children and adolescents, in which LVEF was completely superimposable to healthy normotensive controls. This suggests that impaired LV mechanics can be regarded as an additional marker to target organ damage on top of previously described LVH and LV diastolic dysfunction. These myocardial deformation parameters are important because their impairment occurs before LV diastolic dysfunction and LVH^26^ and represents probably the earliest sign of LV remodeling. Moreover, GLS represents an independent predictor of cardiovascular and total morbidity and mortality in uncomplicated hypertensive patients.^27^ Although this evidence is not currently available for the pediatric population, it is possible to hypothesize a similar trend observed in the adult population.

The most important clinical implication of this meta-analysis is that global longitudinal and circumferential strains were deteriorated in hypertensive children and adolescents with “normal” systolic LV function evaluated using the LVEF metric. Therefore, LV myocardial deformation parameters such as GLS and GCS could be early and sensitive markers for detecting subclinical cardiac damage and monitoring the therapeutic efficacy of antihypertensive therapy. In this regard, unfortunately, very few studies have investigated changes in LV mechanics during antihypertensive treatment and they concern almost exclusively hypertensive adults.^28^To date, evidence on the reversibility of LV mechanical changes due to antihypertensive therapy in the pediatric setting is completely lacking.

Some limitations of the current meta-analysis merit to be mentioned. First, there is heterogenicity of included studies regarding the number of patients, age, and duration of hypertension. Second, studies on LV mechanics in the pediatric setting are very few and therefore our findings cannot be generalized to the whole population of hypertensive children and adolescents. Third, the selected studies used various echocardiographic machines and software for strain evaluation, which might be the source of variation and heterogeneous results among researches.

## Conclusion

Left ventricular remodeling in hypertensive children and adolescents initiates with LV mechanical changes that involve the deterioration of LV longitudinal and circumferential strains. These changes occur even when systolic function assessed with LVEF and FS is fully preserved. Therefore, the early detection of reduced myocardial strain may prompt the initiation or intensification of antihypertensive therapy. The predictive effect of LV strain reduction on outcome in the pediatric setting remains to be evaluated in future longitudinal studies, but results from the adult population reported the significant prognostic impact of reduced LV strain on CV morbidity and mortality in hypertensive patients and there is no reason to question this in younger population.

## Supplementary Material

Supplementary materials are available at *American Journal of Hypertension* (http://ajh.oxfordjournals.org).

hpaf026_suppl_Supplementary_Figures

## Data Availability

Available upon request to the corresponding author
