# Supplementary material for: Elevated Blood Pressure and Cardiac Mechanics in Children and Adolescents: A Systematic Review and Meta-Analysis
Source: Am J Hypertens. 2025 Feb 20;38(6):370–9. doi: 10.1093/ajh/hpaf026 (PMC12080464; doi:10.1093/ajh/hpaf026)
Supplement: hpaf026_suppl_Supplementary_Figures [file hpaf026_suppl_supplementary_figures.zip › Figure S1-S4/Figure S1-S4 Captions.docx]

**Figure S1** Funnel plot assessing publication bias for standardized means difference (SMD) of left ventricular global longitudinal strain (GLS) in healthy normotensive controls and elevated BP/hypertensive individuals.

**Figure S2.** Forest plot of global longitudinal strain (GLS) in healthy normotensive controls and elevated BP/hypertensive individuals (data from six studies including participants aged between 14-18 years). Standard mean difference (SMD) and 95% confidence interval (CI); Random model (I^2^ >75%).

**Figure S3.** Meta-regression of global longitudinal strain (GLS) on systolic blood pressure (BP) in healthy normotensive controls and elevated BP/hypertensive individuals.

**Figure S4.** Meta-regression of left ventricular mass index (LVMI) on systolic blood pressure (BP) in healthy normotensive controls and elevated BP/hypertensive individuals.
