# Supplementary figures and images for: Elevated Blood Pressure and Cardiac Mechanics in Children and Adolescents: A Systematic Review and Meta-Analysis
Source: Am J Hypertens. 2025 Feb 20;38(6):370–9. doi: 10.1093/ajh/hpaf026 (PMC12080464; doi:10.1093/ajh/hpaf026)

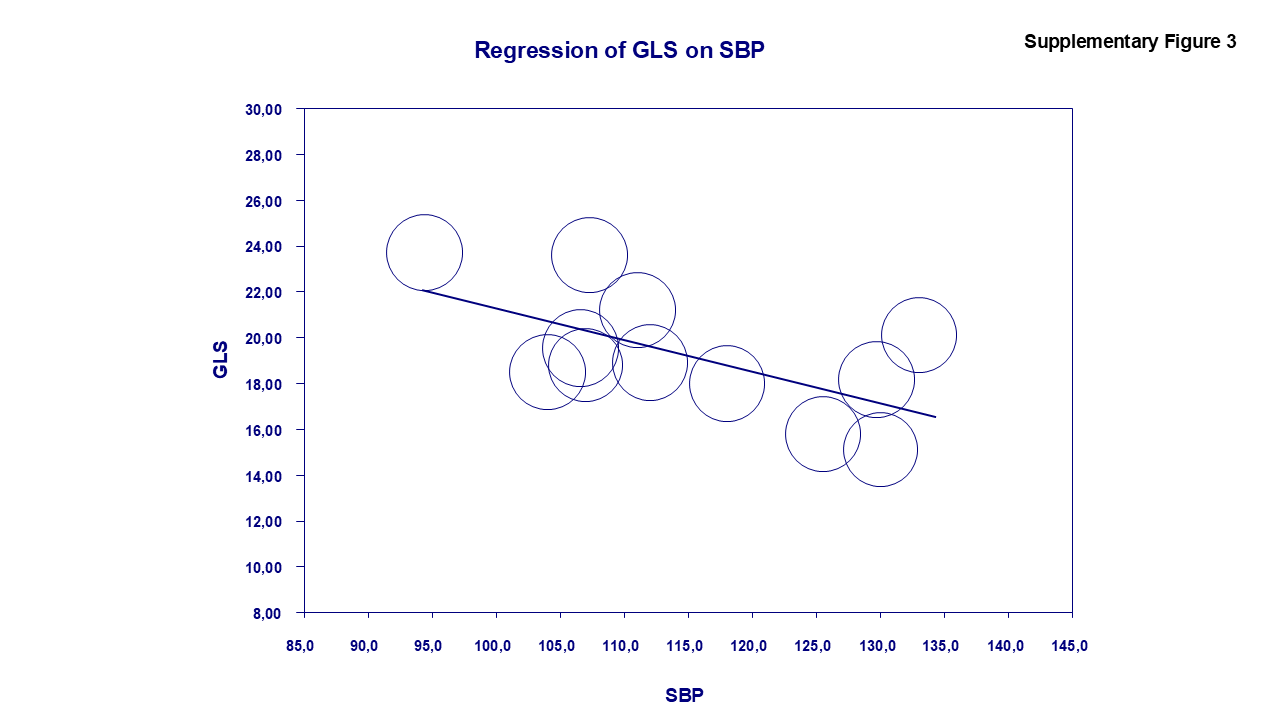

Supplement: hpaf026_suppl_Supplementary_Figures [file hpaf026_suppl_supplementary_figures.zip › Figure S1-S4/Supplementary figure 3 GLS and SBP.tif]

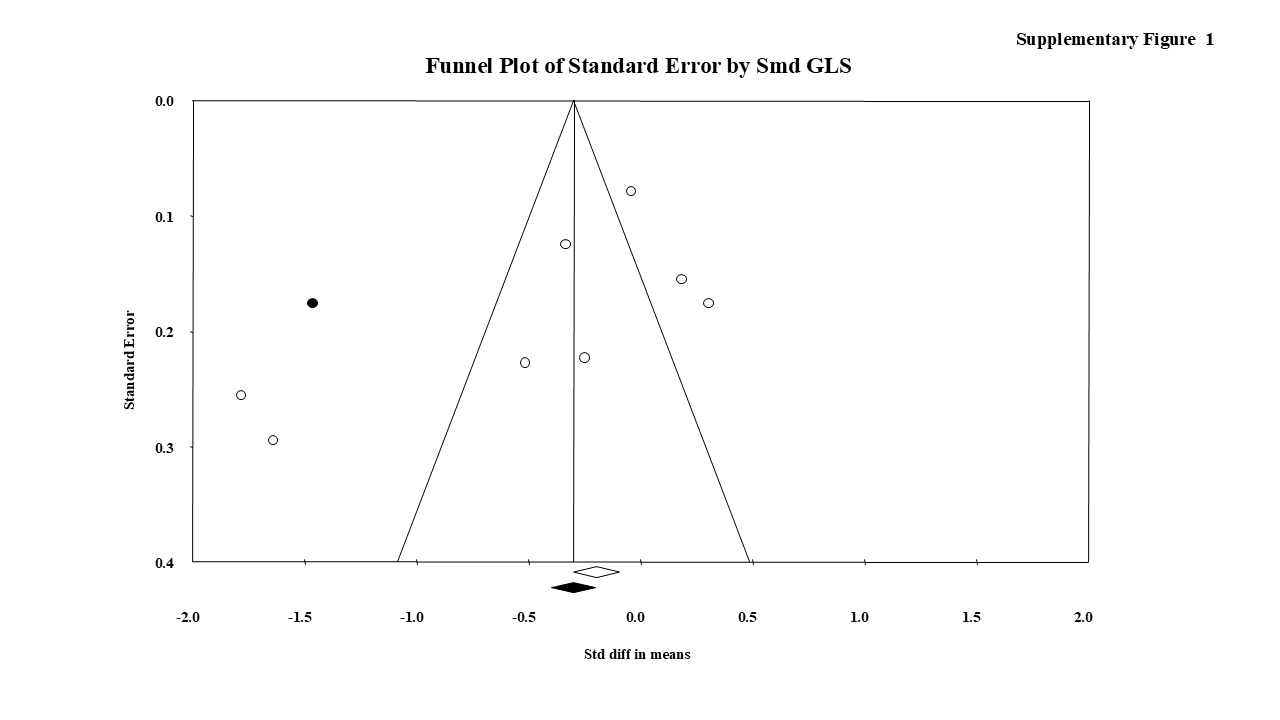

Supplement: hpaf026_suppl_Supplementary_Figures [file hpaf026_suppl_supplementary_figures.zip › Figure S1-S4/Supplementary Figure 1 PB.tif]

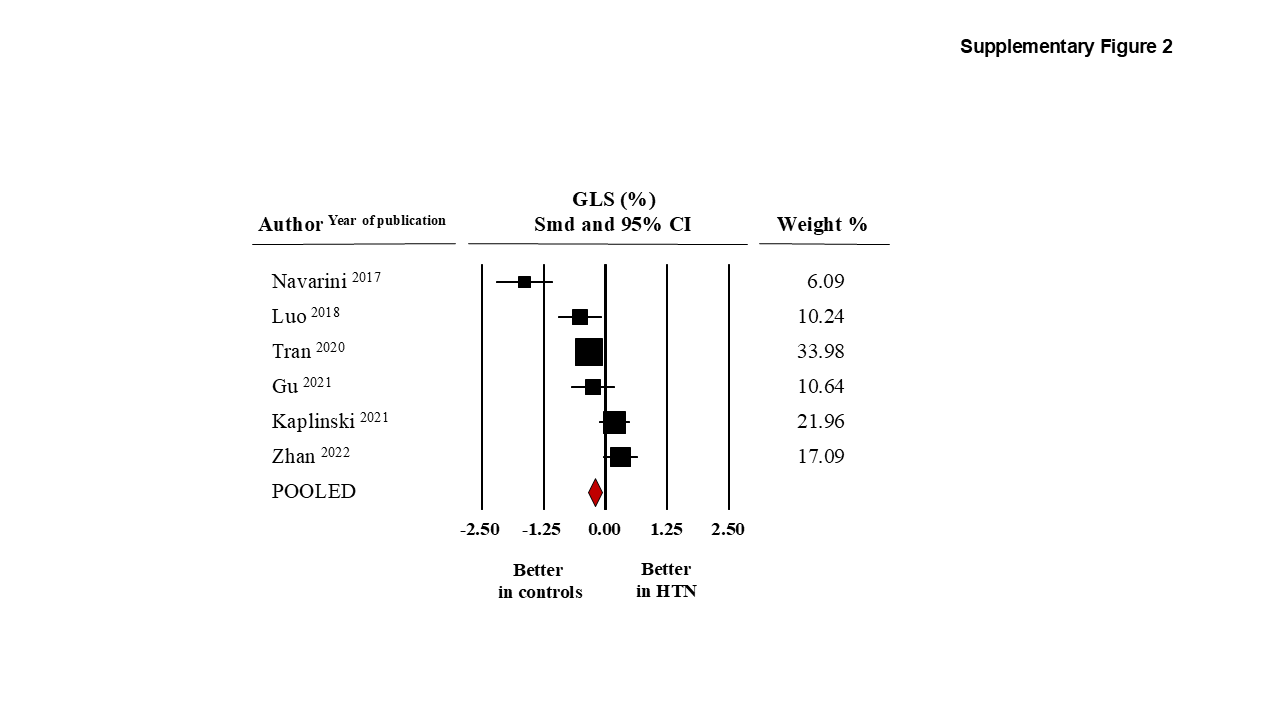

Supplement: hpaf026_suppl_Supplementary_Figures [file hpaf026_suppl_supplementary_figures.zip › Figure S1-S4/Supplementary Figure 2 Forest Plot.tif]

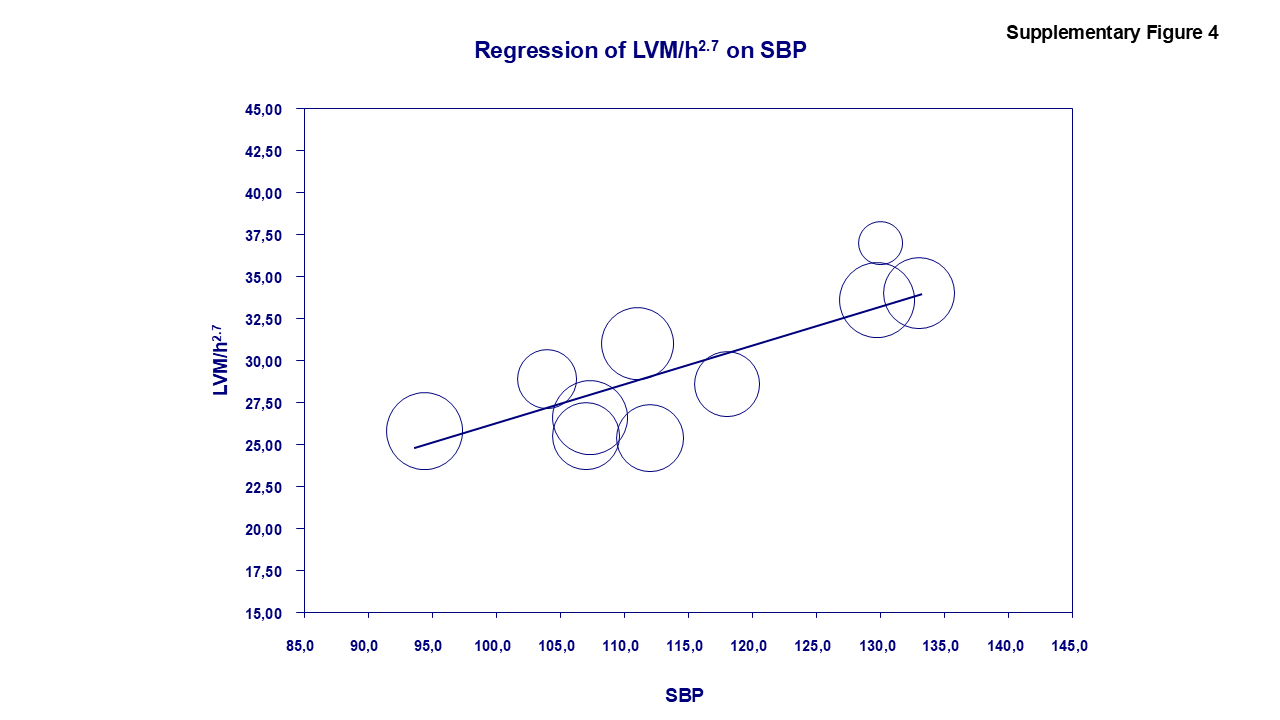

Supplement: hpaf026_suppl_Supplementary_Figures [file hpaf026_suppl_supplementary_figures.zip › Figure S1-S4/Supplementary Figure 4 LVMI and SBP.tif]
